# Tape-*Arabidopsis *Sandwich - a simpler *Arabidopsis *protoplast isolation method

**DOI:** 10.1186/1746-4811-5-16

**Published:** 2009-11-24

**Authors:** Fu-Hui Wu, Shu-Chen Shen, Lan-Ying Lee, Shu-Hong Lee, Ming-Tsar Chan, Choun-Sea Lin

**Affiliations:** 1 Agricultural Biotechnology Research Center, Academia Sinica, Taipei, 115, Taiwan; 2 Scientific Instrument Center, Academia Sinica, Taipei, 115, Taiwan; 3 Department of Biological Sciences, Purdue University, West Lafayette, IN 47907-1392, USA

## Abstract

**Background:**

Protoplasts isolated from leaves are useful materials in plant research. One application, the transient expression of recombinant genes using *Arabidopsis *mesophyll protoplasts (TEAMP), is currently commonly used for studies of subcellular protein localization, promoter activity, and *in vivo *protein-protein interactions. This method requires cutting leaves into very thin slivers to collect mesophyll cell protoplasts, a procedure that often causes cell damage, may yield only a few good protoplasts, and is time consuming. In addition, this protoplast isolation method normally requires a large number of leaves derived from plants grown specifically under low-light conditions, which may be a concern when material availability is limited such as with mutant plants, or in large scale experiments.

**Results:**

In this report, we present a new procedure that we call the Tape-*Arabidopsis *Sandwich. This is a simple and fast mesophyll protoplast isolation method. Two kinds of tape (Time tape adhered to the upper epidermis and 3 M Magic tape to the lower epidermis) are used to make a "Tape-*Arabidopsis *Sandwich". The Time tape supports the top side of the leaf during manipulation, while tearing off the 3 M Magic tape allows easy removal of the lower epidermal layer and exposes mesophyll cells to cell wall digesting enzymes when the leaf is later incubated in an enzyme solution. The protoplasts released into solution are collected and washed for further use. For TEAMP, plasmids carrying a gene expression cassette for a fluorescent protein can be successfully delivered into protoplasts isolated from mature leaves grown under optimal conditions. Alternatively, these protoplasts may be used for bimolecular fluorescence complementation (BiFC) to investigate protein-protein interactions *in vivo*, or for Western blot analysis. A significant advantage of this protocol over the current method is that it allows the generation of protoplasts in less than 1 hr, and allows TEAMP transfection to be carried out within 2 hr.

**Conclusion:**

The protoplasts generated by this new Tape-*Arabidopsis *Sandwich method are suitable for the same range of research applications as those that use the current method, but require less operator skill, equipment and time.

## Background

Plant cells are usually protected by a rigid cell wall comprised of cellulose that provides structural support for the plant. The cell wall can be digested away by an enzyme cocktail containing cellulase and macerozyme, resulting in membrane-bound protoplasts. A variety of different transfection techniques, such as PEG-mediated, electroportation, and microinjection, can then be used to deliver recombinant DNA plasmids into the protoplasts. The transfected protoplasts are then used for further investigations.

In *Arabidopsis *research, protoplast transfection is well established [1-3]. A protocol that has become popular in recent years [4], transient expression in *Arabidopsis *mesophyll protoplasts (TEAMP), uses *Arabidopsis *plants grown in pots as opposed to cells in suspension culture. One advantage of this technique is that it is not necessary to maintain the material in a sterile environment. Also, there is no need to generate suspension cultures, which is time-consuming and often difficult to accomplish with many *Arabidopsis *mutants and ecotypes. There is also an increased likelihood that these protoplasts maintain *in planta *physiology and responses to signal transduction. TEAMP thus has a wide range of applications in plant investigation [4].

The current method of protoplast isolation has several drawbacks that limit its utility. First is the need for strict control of plant growth environment, as some environmental changes will result in reduced transfection efficiency [4]. Because *Arabidopsis *is sensitive to environmental changes, it must be grown under conditions of both relatively short photoperiod and low light for optimal efficiency of TEAMP [4]. The second drawback is the necessity to slice leaves manually. Successful cell isolations require that the leaf is cut into 0.5-1 mm strips, on a suitable support, without crushing the leaf tissue at the cutting site. The blade must be changed after every four or five leaves. A third concern is the length of time required. It takes 4-5 hr to isolate the protoplasts and 6-7 hr to obtain the transfected protoplasts. Therefore, success with the current protocol needs a high degree of skill and much experience.

In tobacco [5], cereals [6], and *Arabidopsis *[7], the leaf epidermal layer can be peeled away from the leaf using forceps with operator skill, so that mesophyll cells can be exposed to the digestion buffer without the epidermis obstructing access. In this report, we describe a simple method that does not suffer from any of the limitations described above. Using two different kinds of tape, the epidermal cells are simply peeled away from the leaf, allowing the enzyme solution access to the intercellular space in order to prepare the protoplasts.

## Methods

### Plant Material and Growth Conditions

*Arabidopsis thaliana *plants were grown in a mixture of vermiculite, perlite, and peat moss at a 1:1:1 ratio in an environmentally-controlled chamber with a long photoperiod (16 hr light and 8 hr dark) at 22°C. The transgenic lines and plasmids were obtained from the Arabidopsis Biological Resource Center (ABRC, Columbus, OH), and had been deposited by Dr. B.K. Nelson [8].

### Protoplast Isolation

Leaves (width: 2 cm, length: 5 cm in optimal light condition; width: 0.5 cm; length: 2.5 cm in low light conditions) were collected from 3 to 5-week-old plants grown under optimal light (ca. 150 μE·m^-2^·s^-1^) or low light (ca. 50·μE m^-2^·s^-1^) conditions. *Arabidopsis *protoplasts were isolated in two ways. First, to recreate the current technique, protoplasts were made according to the procedure of Yoo et al. [4]. Second, in a new technique, selected leaves were used in a 'Tape-*Arabidopsis *Sandwich' experiment. The upper epidermal surface was stabilized by affixing a strip of Time tape (Time Med, Burr Ridge, IL) while the lower epidermal surface was affixed to a strip of Magic tape (3 M, St. Paul, MN). The Magic tape was then carefully pulled away from the Time tape, peeling away the lower epidermal surface cell layer. The peeled leaves (7 to 10 optimal-light-growth leaves, about 1-2 g, up to 5 g), still adhering to the Time tape, were transferred to a Petri dish containing 10 mL of enzyme solution [1% cellulase 'Onozuka' R10 (Yakult, Tokyo, Japan), 0.25% macerozyme 'Onozuka' R10 (Yakult), 0.4 M mannitol, 10 mM CaCl_2_, 20 mM KCl, 0.1% BSA and 20 mM MES, pH 5.7]. The leaves were gently shaken (40 rpm on a platform shaker) in light for 20 to 60 min until the protoplasts were released into the solution. The protoplasts were centrifuged at 100 × *g *for 3 min in an Eppendorff A-4-44 rotor (Hamburg, Germany), washed twice with 25 mL of pre-chilled modified W5 solution (154 mM NaCl, 125 mM CaCl_2_, 5 mM KCl, 5 mM glucose, and 2 mM MES, pH 5.7) and incubated on ice for 30 min. During the incubation period, protoplasts were counted using a hemocytometer under a light microscope. The protoplasts were then centrifuged and resuspended in modified MMg solution (0.4 M mannitol, 15 mM MgCl2, and 4 mM MES, pH 5.7) to a final concentration of 2 to 5 × 10^5 ^cells/mL.

### Protoplast Transfection Assays

Protoplasts were transfected by a modified TEAMP method [4]. Approximately 5 × 10^4 ^protoplasts (2 × 10^4 ^to 1 × 10^5^) in 0.2 mL of MMg solution were mixed with approximately 30 (20 to 40) μg of plasmid DNA at room temperature. An equal volume of a freshly-prepared solution of 40% (w/v) PEG (MW 4000; Fluka) with 0.1 M CaCl_2 _and 0.2 M mannitol was added, and the mixture was incubated at room temperature for 5 min. After incubation, 3 mL of W5 solution was added slowly, the solution was mixed, and protoplasts were pelleted by centrifugation at 100 × *g *for 1 min (Eppendorff A-4-44 rotor). This protoplast W5 wash step was repeated twice. The protoplasts were resuspended gently in 1 mL of W5 and were incubated in 6-well plates coated with 1% BSA at room temperature for 16 hr in light.

### Confocal Laser Scanning Microscopy

Protoplasts were observed with a Zeiss LSM510 META laser scanning confocal microscope using 20×/0.8 Plan-Apochromat, 40×/1.2 W C-Apochromat or 63×/1.4 Oil Plan-Apochromat in multi-track channel mode. Excitation wavelengths and emission filters were 488 nm/band-pass 505-530 nm for GFP or YFP, 458 nm/band-pass 465-530 nm for CFP, and 488 nm/band-pass 650-710 nm for chloroplast auto-fluorescence. The images are presented as 3D projected stacks of neighboring sections as stated in the figure legends. Image processing was performed using LSM 510 version 4.2 (Zeiss), and the included three-dimensional reconstruction functions were used to compute projections of serial confocal sections.

### Light Microscopy

Tissue was fixed in 2% glutaraldehyde in 25 mM sodium phosphate buffer at pH 6.8, vacuum infiltrated, and incubated at 4°C overnight. Tissue was briefly rinsed in 25 mM sodium phosphate buffer, pH 6.8, and dehydrated in an ethanol series (25, 50, 70, 95, and 100%), with 2 hr in each solution. Then the tissue was incubated through a series of LR white resin/ethanol mixes (25:75, 50:50, 75:25, 100:0 two times) and finally embedded in LR white resin. Using a microtome, 2-μm cross sections were cut and placed on glass slides. These were stained by periodic acid Schiff (PAS) reaction, then water mounted and covered with a cover slip for observation by light microscopy.

### Western Blot

Proteins were extracted by homogenizing 10^5 ^protoplast cells in 50 μL of 1× lysis buffer (Promega, Madison, WI). Recombinant γGFP (Roche, Mannheim, Germany) was used as a positive control and for quantification. Proteins were separated by SDS-polyacrylamide gel electrophoresis (SDS-PAGE) and then transferred onto a nitrocellulose membrane (Millipore, Billerica, MA). The membrane was incubated first with mouse anti-green fluorescent protein antibody (Roche) and then with rabbit anti-mouse IgG-alkaline phosphatase conjugates (Sigma). The blot was developed using BCIP/NPT Color Development Substrate (Sigma). Immunoblots were digitized and the intensity of the bands measured with Multi Gauge software (Fujifilm, Tokyo, Japan).

## Results and Discussion

### A simple method for protoplast isolation

The current standard *Arabidopsis *mesophyll protoplast isolation protocol is well-established, and has also been used in various modified forms for specific needs [4]. In this protocol, five-week-old plants are used for protoplast isolation. To reduce the time taken from seed to generate protoplasts, 14-d-old plantlets have also been used for protoplast isolation [9]. However, this method consumes many seeds, which is a disadvantage for the study of mutants that have a low seed production phenotype. This report presents an alternative simple, fast, protoplast isolation method for TEAMP that we have named the Tape-*Arabidopsis *Sandwich method, where material availability poses far less of a limitation.

**Figure 1:**
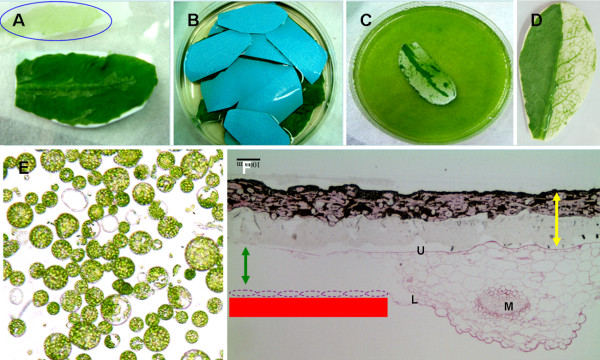
**Using Tape-*Arabidopsis *Sandwich for *Arabidopsis *protoplast isolation**. (A) The upper epidermis was stuck onto Time tape and the lower epidermis was stuck to clear 3 M tape. The lower epidermal layer was removed by peeling away the 3 M tape; the blue circle indicates cells from the lower epidermis on the 3 M tape after peeling. (B) The leaves with Time tape still adhering to the top surface (with blue backing) were incubated in enzyme solution. (C) After 1 hr, the cell walls were digested and the protoplasts were released into the bottom of the dish. (D) The enzymes only digested the cell wall of those mesophyll cells that were not protected by lower epidermal cells. Left: intact; right: lower epidermal cells removed. (E) Protoplasts derived from mature Arabidopsis leaves. (F) After incubation in the enzyme solution, all mesophyll cells became protoplasts: microscopy showed that only upper epidermal cells remained. Yellow arrow: Time tape; red line: the relative position of 3 M Magic tape where the lower epidermal cell layer (purple circle) was removed; green arrow; the related position of mesophyll. M: midrib; U: upper epidermis; L: lower epidermis. Bar = 100 μm.

For the Tape-*Arabidopsis *Sandwich method (see methods for full description) clear 3 M tape was used to peel away the lower epidermal cell layer (Fig. 1A) from 7 to 10 leaves whose upper epidermis was adhered to a strip of Time tape. Peeled leaves were transferred to enzyme solution (Fig. 1B, C). The enzyme mixture only digested the cell walls from mesophyll cells that was not protected by the epidermis, as demonstrated by using tape to remove the epidermal layer on only one half of a leaf (Fig. 1D). Microscopy confirmed the release of protoplasts into solution (Fig. 1E). Because all of the mesophyll cells became protoplasts, microscopy showed that only upper epidermal cells remained (Fig. 1F). There is no mechanical cutting step in this protocol; the protoplast suspension did not need to be filtered to remove any undigested tissues.

**Figure 2:**
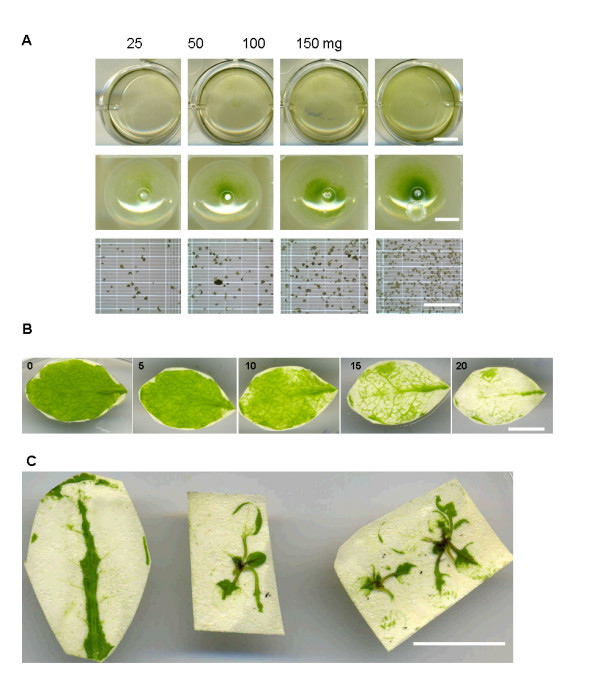
**Tape-*Arabidopsis *Sandwich method generates sufficient protoplasts for transient transfection and leaves of any stage can be used**. A. Different weights of leaves (25, 50, 100, 150 mg) were incubated in 2 ml digestion buffer (row 1), protoplasts were collected (row 2) and 1.20 × 10^4^, 3.15 × 10^4^, 6.84 × 10^4^, and 5.49 × 10^5 ^protoplasts were obtained (row 3). Bar: row 1 and 2 = 0.5 cm, row 3 = 0.5 mm. B. Arabidopsis leaves incubated in digestion buffer for 0, 5, 10, 15, 20 min. The protoplasts were fully released within 30 min. Bar = 1 cm. C. Both mature leaf (left) and two week-old seedlings (middle and right) could be used for protoplast isolation. Bar = 1 cm.

Using this method, enough protoplasts (1.2 × 10^4^, Fig 2A) for one transfection event were generated from a small amount of tissue (25 mg fresh-weight leaves). An average of 3.0 ± 0.3 × 10^7 ^protoplasts were isolated from 1 g leaves in 30 mL digestion buffer (*n *= 3; Table 1) in 20 min to 1 hr (Fig. 2B). In comparison with the cutting method, this tape method generated sufficient protoplast numbers for subsequent experimental use in much less time.

**Table 1:** Effects of plant material on protoplast isolation on protoplast transfection.

Light condition	Method	Protoplast No. (×10^**7**^)	Transformation efficiency (%)*	Broken (%)
Low	Cutting	1.8 ± 0.3	64.8 ± 5.8	1.7 ± 2.9
Low	Tape	1.4 ± 0.2	66.2 ± 8.4	3.2 ± 5.6
Optimal	Cutting	3.7 ± 1.3	53.0 ± 4.0	24.9 ± 10.3
Optimal	Tape	3.0 ± 0.3	60.7 ± 3.2	1.8 ± 3.3

One gram of leaves derived from plants was grown under normal (optimal) or low-light conditions [4] were used. Protoplasts were isolated and purified using Tape-*Arabidopsis *Sandwich and cutting protocols. Total cell number (intact and broken cells) was calculated. The 'Broken (%)' is: 100 × (broken cell number/total cell number). The broken protoplasts were not counted during the transfection efficiency calculation. The transfection efficiency is the percentage of intact protoplasts with fluorescent/intact protoplasts. These protoplasts were transformed with VirE2-Cerullean. Each treatment was replicated three times (*n *= 3).

*: % of intact protoplasts showing VirE2-Cerulean fluorescence.

Tape can be used to peel off the epidermis of leaves at any stage of development, from seedlings to mature leaves (Fig. 2C). This protocol is therefore highly advantageous when there are limitations imposed by material availability following specific treatments or in growth-retarded mutants. Not only can any size of leaf be used, but also the plants do not need to be grown under specific conditions, such as low-light conditions. When using the current standard protocol, changes in growth conditions have been reported to affect transfection efficiency [4]. There was no significant difference in transfection efficiency between optimal light and low light conditions in protoplasts isolated by the Tape-*Arabidopsis *Sandwich method (Table 1). The ability to grow plants under optimal conditions and thus obtain a higher biomass for protoplast isolation will be of much benefit for large scale experiments.

**Figure 3:**
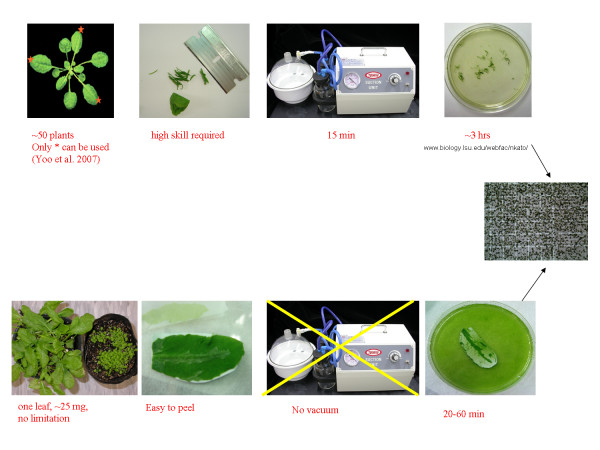
**Protoplast isolation flowchart for the cutting method (upper) and the Tape-*Arabidopsis *Sandwich method (lower)**.

Because there is no cutting in this protocol, less damage is done to the leaf tissue when using the plants grown under optimal conditions (Table 1). In addition, there is no need for a vacuum filtration process (another cause of damage to protoplasts) as all of the non-digested residual tissue remains stuck on the tape, and only the protoplasts are released into the digestion solution. The fact that undigested material is not released into in the digestion solution means that the filtering or sugar gradient purification step can be eliminated. Routinely, the whole process takes less than 3 hr, including the protoplast isolation (1 hr, Fig. 3) and transfection procedures (2 hr).

### Suitability of protoplasts for study of subcellular localization and protein-protein interactions

The imaging of various fluorescent proteins fused to functional proteins is the method of choice for the study of subcellular localization [4]. Genes encoding fluorescent-tagged proteins such as organelle markers can be transfected into protoplasts for transient expression or into *Arabidopsis *germ cells to develop stably-transfected plants. The subcellular localization of a specific tagged protein can be examined by fluorescence microscopy. There are many fluorescent organelle marker systems employing proteins of known localization [8,10,11]. In this report, we used the system of Nelson et al. [8].

**Figure 4:**
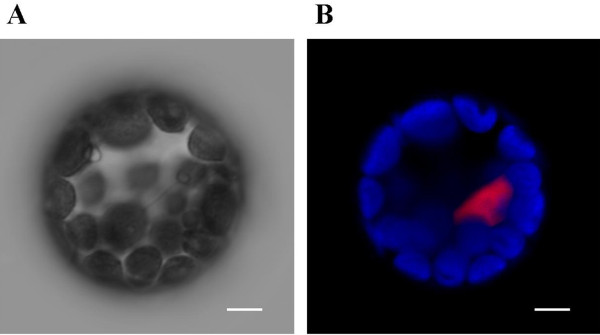
**Plasmids can be transferred to sandwich-method protoplasts for transient expression of recombinant genes**. The plasmid containing VirD2-NLS --mCherry was transferred into protoplasts by the PEG method. (A) DIC images, (B) Fluorescence images; bar = 5 μm.

We used a PEG-mediated method (TEAMP) to deliver plasmids encoding fluorescent organelle marker proteins into protoplasts. The fluorescence was always detected in the predicted organelle of the transfected protoplast. As an example, we transfected VirD2-NLS-mCherry [12] into protoplasts and achieved a satisfactory transfection efficiency using TEAMP (Fig. 4).

**Figure 5:**
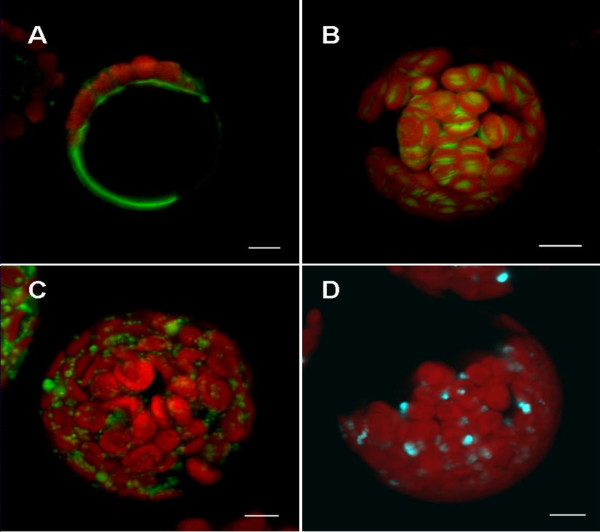
**Characteristic features of organelle markers in mesophyll-derived protoplasts of stably transformed lines of *Arabidopsis***. (A) YFP vacuole marker: γ-TIP, an aquaporin of the vacuolar membrane. ABRC stock no.: CS16258. (B) GFP plastid marker: the targeting sequence (first 79 aa) of the small subunit of tobacco Rubisco fused with GFP. ABRC stock no.: CS16266. (C) YFP mitochondria marker: first 29 amino acids of yeast cytochrome c oxidase IV fused with YFP. ABRC stock no.: CS16264. (D) CFP peroxisome marker: cytoplasmic tail and transmembrane domain of soybean -1,2-mannosidase I fused with CFP [8]. ABRC stock no.: CS16259. Bar = 5 μm.

We also tested the protoplast isolation method reported here using leaves from different stable transfection lines (Fig. 5). The tonoplast marker (γ-TIP-YFP, Fig. 5A) appeared in the predicted organelles. In the chloroplast GFP marker line (using the targeting sequence of the small subunit of tobacco Rubisco), all of the chloroplasts in the protoplasts had yellow fluorescence (Fig. 5B). The YFP mitochondria-marker line showed the fluorescence in the mitochondria of isolated protoplasts (Fig. 5C). The peroxisomes were identified by fluorescence in peroxisome-marker (PTS2, Ser-Lys-Leu) CFP lines (Fig. 5D).

**Figure 6:**
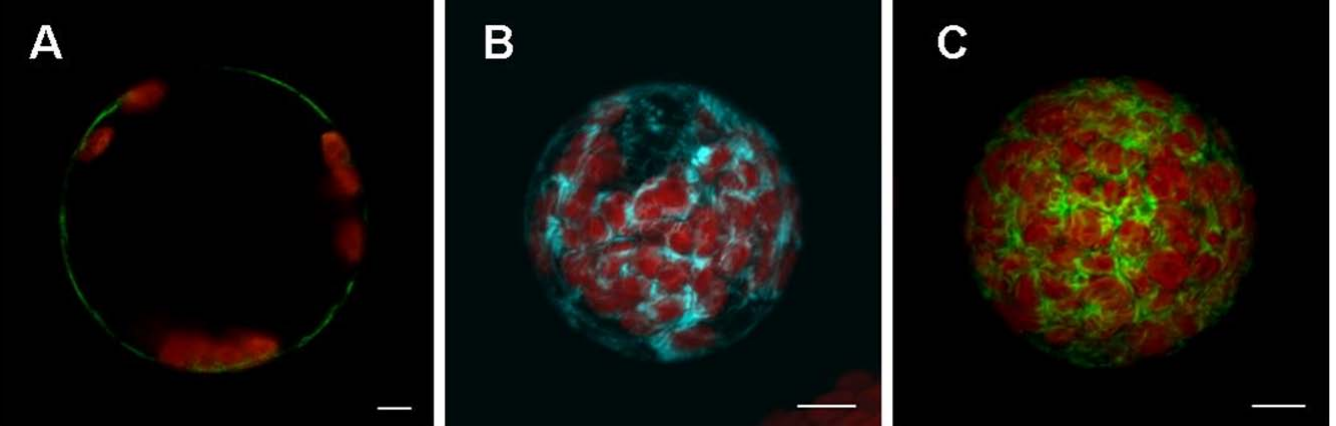
**Plasmids encoding a variety of fluorescent proteins may be successfully delivered to protoplasts**. Protoplasts transiently transfected with (A) YFP plasma membrane marker, the full length of AtPIP2A, a plasma membrane aquaporin, ABRC stock no.: CD3-1005, (B) CFP (ABRC stock no.: CD3-953), and (C) a YFP-tagged ER marker (ABRC stock no.: CD3-957), the signal peptide of AtWAK2 (*Arabidopsis thaliana *wall-associated kinase 2) at the N-terminus and the ER retention signal at C-terminus of the fusion protein [8]. Transfections used the PEG-mediated method. Bar = 5 μm.

**Figure 7:**
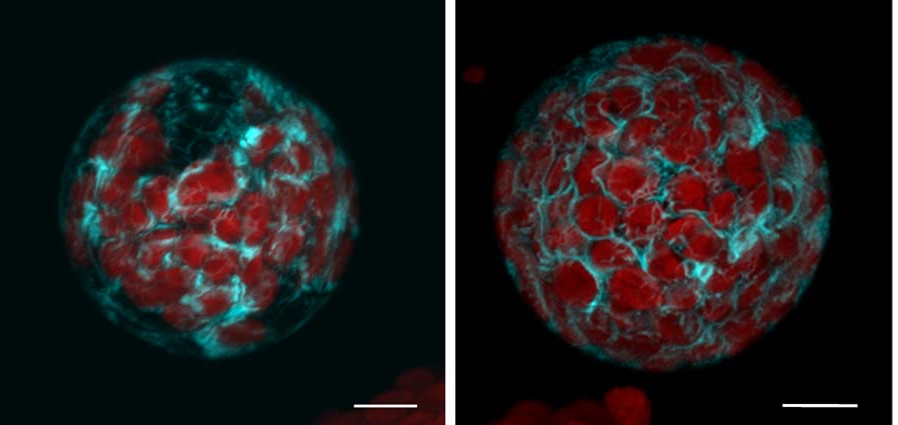
**The tape sandwich method does not compromise expression patterns**. A CFP-tagged ER marker (ABRC stock no.: CD3-953), the signal peptide ofAtWAK2 at the N-terminus of the FP, and the ER retention signal at C-terminus [8], was delivered into wild-type protoplasts by transient transfection (left). Protoplasts derived from stable transformation with the same plasmid (right, ABRC stock no.: CS16250) have the same expression pattern.

Maintaining the stable fluorescent protein marker lines takes space and manpower and is not convenient for many studies. Therefore, our main aim was to perform direct transient transfection. Although the plasmids used were larger than 10 kb, the plasma membrane YFP marker (Fig. 6A) and ER marker plasmid (His-Asp-Glu-Leu fused to C-terminus of FP; Fig. 6B and 6C) were successfully transfected into protoplasts, and the resulting protoplasts correctly expressed and synthesized the fluorescent protein. With an ER marker, the expression patterns in stable lines obtained from the ABRC and transient expression in wild-type *Arabidopsis *protoplasts were similar (Fig. 7). These markers did not show altered their subcellular localization when transiently expressed in protoplasts compared to protoplasts isolated from transgenic plants expressing the identical marker proteins.

Many proteins will form a complex with other protein molecules in order to elaborate or modify their function. There are many methods developed to investigate protein-protein interactions. One of these visualization methods, bimolecular fluorescence complementation (BiFC), can observe such interactions in living cells [12-16]. BiFC works by fusing two non-fluorescent halves (N-terminal and C-terminal) of a fluorescent protein to two interacting proteins such that interaction leads to restoration of fluorescence within the cell [17].

**Figure 8:**
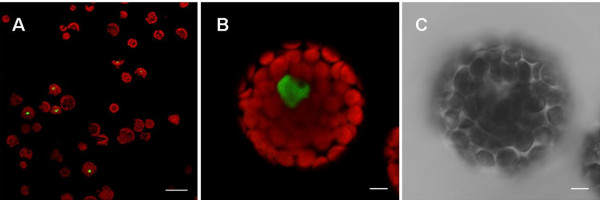
**BiFC visualization of bZIP63 dimerization in transfected *Arabidopsis *protoplasts**. Epifluorescence (A, B) and DIC (C) images of protoplasts that were transfected with plasmids encoding fusion proteins. Green color indicates the fluorescence as a result of bZIP63 dimerization in BiFC. The dimer of bZIP63 was located in the nucleus [14]. Bar in A = 50 μm, bar in B and C = 5 μm.

In this report, we used the positive control previously reported by Walter et al. [14]. These plasmids encode the bZIP63 fusion with N-terminus and C-terminus of GFP. The bZIP proteins interact to form a homodimer, located in the nucleus. This phenomenon has been demonstrated in yeast two-hybrid experiments, in an *Arabidopsis *cell culture protoplast transient expression system, and in *Agrobacterium*-infiltrated tobacco leaves [14]. This interaction could also be observed in protoplasts derived from mesophyll cells following the new protocol presented in this report (Fig. 8).

**Figure 9:**
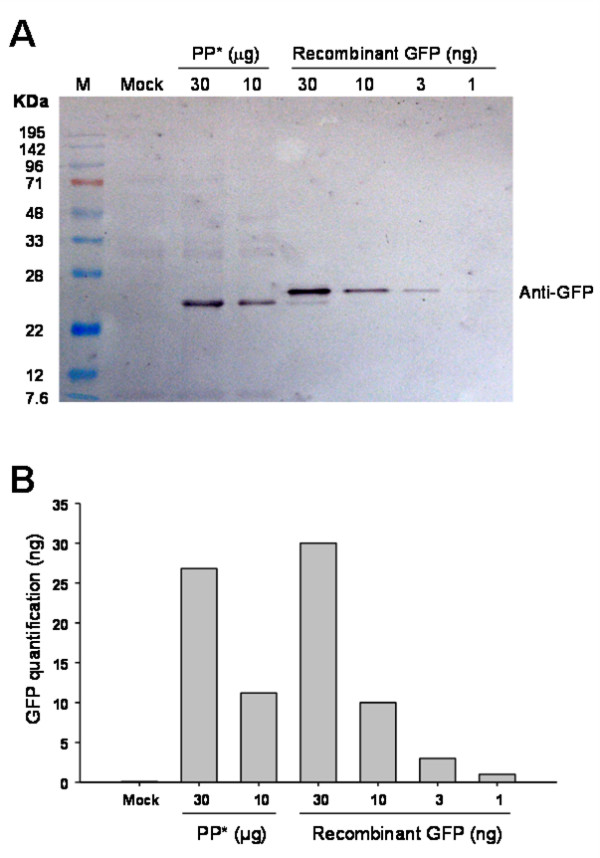
**Transfected protoplast protein was isolated for Western blot assay**. (A) Protoplasts were transfected with a 35S- soluble-modified GFP plasmid (MW: 26.7 kDa). Protoplast protein (lane 3 and 4) was extracted 16 hr later and the expressed proteins were analyzed by SDS-PAGE followed by Western blotting with anti-GFP antibody. Recombinant γGFP (Roche, Mannheim, Germany; lane 5-8, 26.8 kDa) was used for positive control and quantification. (B) Immunoblots were digitized and the intensity of the bands measured with Multi Gauge software (Fujifilm, Tokyo, Japan). About 0.1% of the total protein of protoplast was GFP. M: marker; PP*: transfected protoplast protein.

Proteins from transfected protoplasts can be extracted for further analyses such as Western blot (Fig. 9). Using the CaMV 35S promoter-GFP construction, we found in our example that about 0.1% of total protein was derived from the transfection plasmid. These results indicate the protoplasts derived from Tape-*Arabidopsis *Sandwich can constitute useful plant material for further investigation.

## Conclusion

The modified protoplast isolation method, Tape-*Arabidopsis *Sandwich, presented here allows mesophyll cells to be exposed to a cell wall digesting enzyme solution without macerating the cells. The enzyme solution has much easier access to the intercellular space than in the previous method. Small amounts of leaf tissue (1-2 g, about 6-7 fully expanded leaves) are enough to yield about 10^7 ^protoplasts in 1 hr, and these cells are ready for transient transfection within 2 hr. We have also demonstrated here that protoplasts obtained from this protocol can be used for efficient transient transgene expression, localization of different organelle markers, and in BiFC for protein-protein interaction analysis. We believe that this protoplast isolation method could have wide applications and be adopted by many plant researchers.

## Competing interests

The authors declare that they have no competing interests.

## Authors' contributions

FHW performed all transfections. SCS performed confocal microscope observation. SHL and MTC performed Western blot. LYL participated in preparation of the manuscript. CSL conceived this project, supervised protoplast isolation and transfection and participated in preparation of the manuscript.
